# Co-carcinogenic effects of vitamin E in prostate

**DOI:** 10.1038/s41598-019-48213-1

**Published:** 2019-08-12

**Authors:** Fabio Vivarelli, Donatella Canistro, Silvia Cirillo, Alessio Papi, Enzo Spisni, Andrea Vornoli, Clara M. Della Croce, Vincenzo Longo, Paola Franchi, Sandra Filippi, Marco Lucarini, Cristina Zanzi, Francesca Rotondo, Antonello Lorenzini, Silvia Marchionni, Moreno Paolini

**Affiliations:** 1 Department of Pharmacy and Biotechnology, University of Bologna, Bologna, Italy; 2 Department of Biological, Geological and Environmental Sciences, University of Bologna, Bologna, Italy; 3 Cesare Maltoni Cancer Research Center (CMCRC), Ramazzini Institute (RI), Bentivoglio, Bologna, Italy; 4 Department of Chemistry “G. Ciamician”, University of Bologna, Bologna, Italy; 5 Department of Agricultural Biology and Biotechnology, CNR, Pisa, Italy; 6 Interdepartmental Laboratory of Functional and Cellular Pharmacology of Reproduction, Department of Neuroscience, Drug Research and Child Care, University of Florence, Florence, Italy; 7 Center for Environmental Toxicology, Environmental Protection and Health Prevention Agency Emilia-Romagna Region (ER-EPA), Bologna, Italy; 8 Center for Environmental Toxicology, Agency for Prevention, Environment and Energy, Emilia-Romagna, Bologna, Italy; 9 Department of Biomedical and Neuromotor Sciences, University of Bologna, Bologna, Italy

**Keywords:** Risk factors, Prostate

## Abstract

A large number of basic researches and observational studies suggested the cancer preventive activity of vitamin E, but large-scale human intervention trials have yielded disappointing results and actually showed a higher incidence of prostate cancer although the mechanisms underlying the increased risk remain largely unknown. Here we show through *in vitro* and *in vivo* studies that vitamin E produces a marked inductive effect on carcinogen-bioactivating enzymes and a pro-oxidant status promoting both DNA damage and cell transformation frequency. First, we found that vitamin E in the human prostate epithelial RWPE-1 cell line has the remarkable ability to upregulate the expression of various phase-I activating cytochrome P450 (CYP) enzymes, including activators of polycyclic aromatic hydrocarbons (PAHs), giving rise to supraphysiological levels of reactive oxygen species. Furthermore, our rat model confirmed that vitamin E in the prostate has a powerful booster effect on CYP enzymes associated with the generation of oxidative stress, thereby favoring lipid-derived electrophile spread that covalently modifies proteins. We show that vitamin E not only causes DNA damage but also promotes cell transformation frequency induced by the PAH-prototype benzo[a]pyrene. Our findings might explain why dietary supplementation with vitamin E increases the prostate cancer risk among healthy men.

## Introduction

Prostate cancer is the most common human malignancy and the second leading cause of cancer death among men in western nations. The high global incidence of prostate cancer, the unsatisfactory outcomes of surgery and radiotherapy and the cost of curative therapies have led to a focus on primary prevention as a major public health goal^1,2^. Supported by preclinical and epidemiological evidence, antioxidants from food and supplements are widely used to protect against cancer, but clinical trials do not sustain this concept^1,3–6^ and actually showed a higher incidence of prostate cancer^7,8^. Conceived to break through this issue, in 2001 the National Cancer Institute (NCI) launched SELECT (Selenium and vitamin E Cancer Prevention Trial), that showed a 17% increase in prostate cancer incidence in the vitamin E arm compared to placebo^7^.

A growth-promoting effect of vitamin E on organoids has recently been reported^9^, but its mechanism of action remains poorly understood. Since some cytochrome P-450 (CYP) isoforms have been found overexpressed in prostate cancer^10–15^, we suspected that vitamin E could have co-carcinogenic properties such as those involving carcinogen-bioactivating CYP-enzyme changes^16,17^.

## Results and Discussion

**Figure 1:**
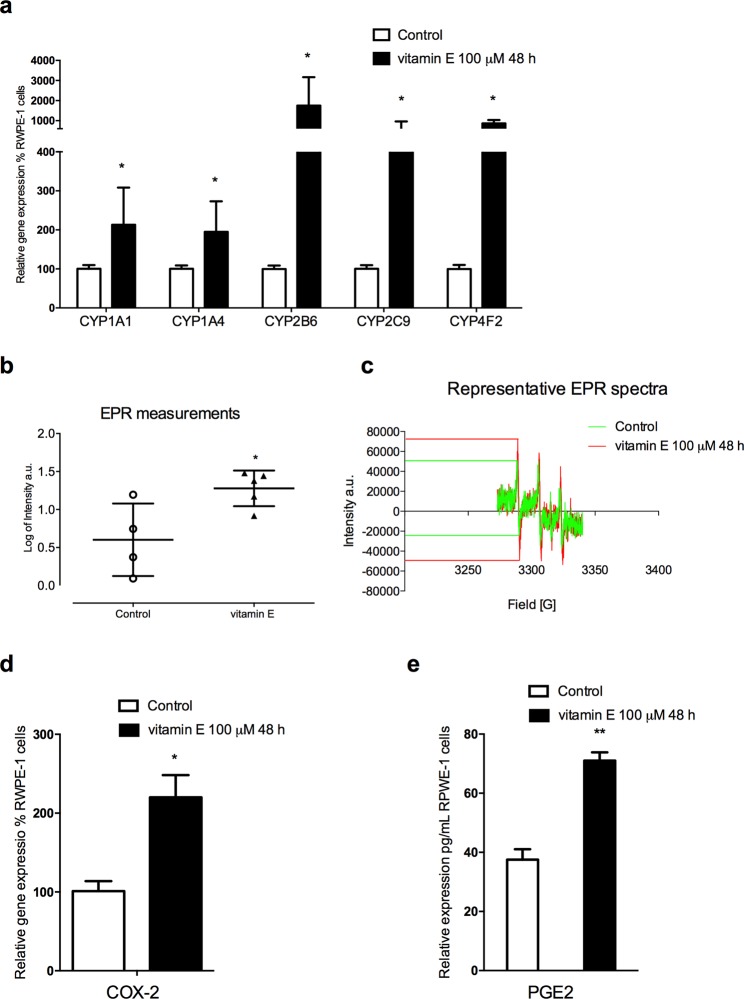
Vitamin E in RWPE-1 cell line induces CYP gene expression coupled with increased ROS release and inflammation markers. (**a**) Gene expression study on RWPE-1 cells revealed sharp increments in mRNA levels from vitamin E-exposed cells compared to control. CYP1A1 and CYP1A4 are doubled (*P* < 0.05; *n* = 3). Isoforms 2B6, 2C9 and 4F2 showed the most pronounced changes with 18, 4 and 7 fold-higher levels respectively (*P* < 0.05; *n* = 3). (**b**) Free radical measurements through EPR spectroscopy in RWPE-1 cell line. The scattered dot plot represents total oxygen, nitrogen, and carbon-centered free radical species (arbitrary units) measured in RWPE-1 cells 48 hours after vitamin E exposure. Exposed cells (*n* = 5) exhibited significantly higher levels of radical species compared to controls (*n* = 4; *P* < 0.05). (**c**) Representative EPR spectra of nitroxide radicals observed in RWPE-1 control samples (green spectra), and in vitamin E-exposed samples (red spectra). EPR intensity of the first spectral line was used to obtain the relative amount of nitroxide in each sample examined. (**d**) Vitamin E-exposed cells (100 μM; 48 h) exhibited higher COX-2 mRNA levels (*P* < 0.05; *n* = 3). (**e**) COX-2 mRNA induction was coupled with an increased PGE2 secretion (*P* < 0.01; *n* = 3). Each bar represents the mean (±SD) *P* < 0.05, **P < 0.01 two-tailed t-test. (Control *vs* groups of each treatment).

Our study stemmed from the observation that vitamin E treatment of the human non-tumorigenic prostate epithelial RWPE-1 cell line induces the gene expression of P450 enzymes such as CYP1A1 (activating, for example, polychlorinated biphenyls, aromatic amines, PHAs and alkylnitrosamines), CYP1A4 (activating polycyclic aromatic hydrocarbons, PAHs), CYP4F2 (the major enzyme involved in vitamin E metabolism), CYP2C9 (activating heterocyclic aromatic amines and PAHs), and an up to 18-fold increase in the expression of CYP2B6 (activating tobacco smoke pro-carcinogens such as 4-methylnitrosamino-1-3-pyridyl-1-butanone, NNK) (Fig. 1a). These results suggested that vitamin E enhances the bioactivating potential of the CYP supergene family, making prostate cells more vulnerable to exposure to pro-mutagenic and pro-carcinogenic agents. It is noteworthy that a meta-analysis revealed that CYP1A1 polymorphisms are closely related to the prostate cancer risk^11^, and that CYP1A1 and CYP1B1 are overexpressed in prostate cancer cells^10–13^. Conversely, reduced basal CYP1A1 mRNA expression leads to an inhibition of cell proliferation by inducing apoptosis in prostate cancer cells^12^. These data support the hypothesis that vitamin E could contribute to prostate carcinogenesis via CYP mediated mechanism.

We then used electron paramagnetic resonance (EPR) to evaluate the exact contribution of vitamin E-induced CYPs to superoxide radical production, finding that vitamin E in RWPE-1 prostate cells stimulates the production of supraphysiological levels of reactive oxygen species (ROS) (Fig. 1b,c) primarily derived by uncoupling the CYP-Fe II-O_2_ complex of the CYP catalytic cycle^18,19^. These data provide evidence of the dual nature of vitamin E, a two-edged sword acting directly as an antioxidant and indirectly as a pro-oxidant^20^. In addition, vitamin E plays a critical role in impairing redox homeostasis, while the pro-inflammatory cyclooxygenase-2 (COX-2) prostaglandin-2 (PGE2) pathway is involved in various malignancies, including prostate cancer^21,22^, by means of resistance to apoptosis, increased proliferation, invasiveness, metastasis and angiogenesis. These findings are currently fuelling research focused on COX-2 inhibitors as an anti-cancer strategy^23,24^, so we performed experiments to detect these markers. We observed that vitamin E enhances the expression of COX-2 mRNA which, in turn, yields overproduction of its major metabolite PGE2 (Fig. 1d,e). These data reinforce the hypothesis of vitamin E’s role in growth-promoting prostate tumours, although, it should be taken into account that the concentration here tested is supraphysiological and higher compared to the circulating levels achieved in subjects enrolled in the SELECT study^25^.

**Figure 2:**
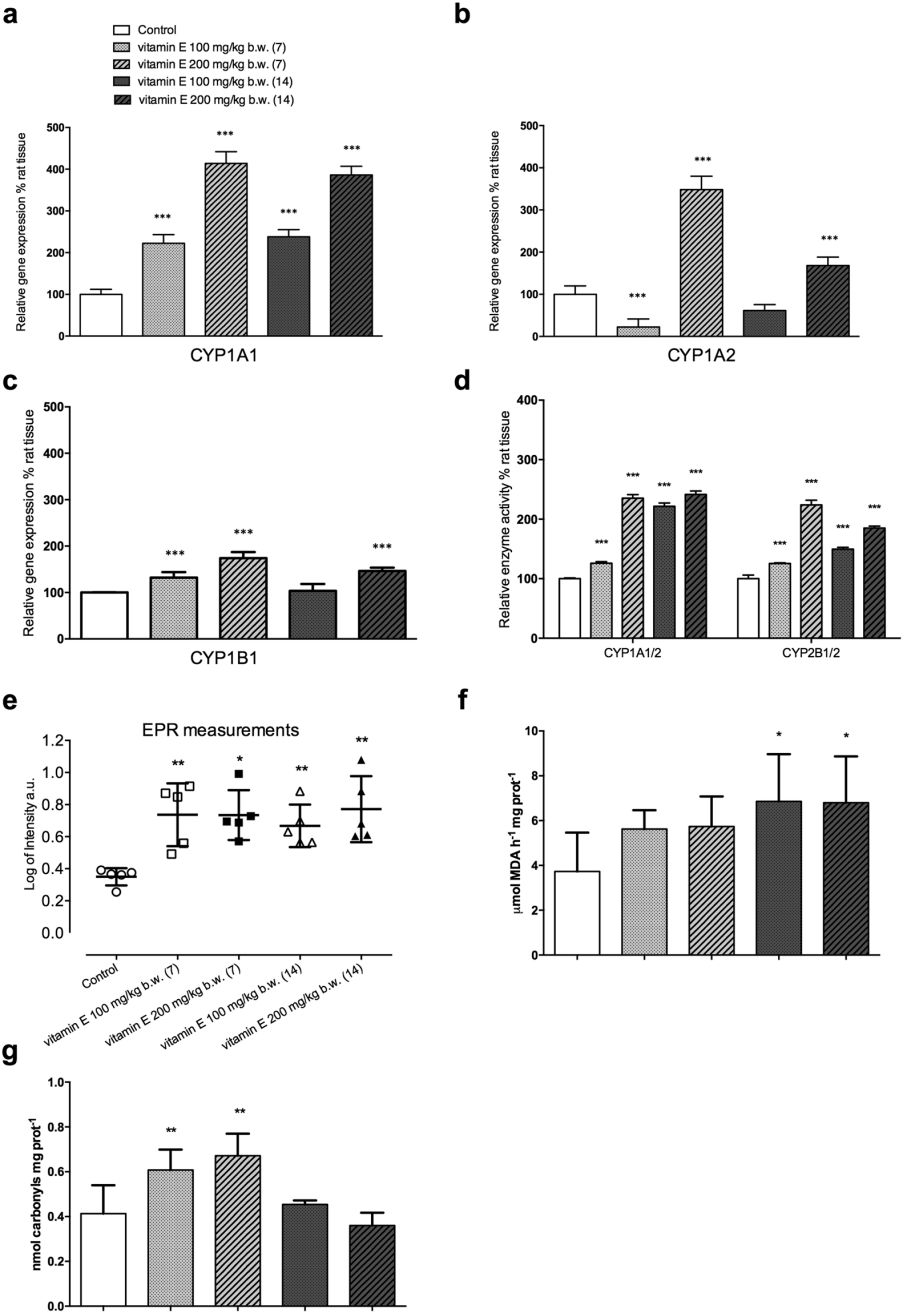
Vitamin E in rat prostate boosts CYP gene expression and linked enzymatic activities, and impairs the redox imbalance. (**a**) Data from RT-PCR analysis reported a marked increment of CYP1A1 throughout treatment (*P* < 0.001; *n* = 5). (**b**) CYP1A2 presented a non-linear pathway of modulation with a significant inhibition at the lowest dose (7 days) that was not confirmed after 14 days. 200 mg/kg b.w. led to a net upregulation at both time windows. (**c**) CYP1B1 showed a trend similar to that described for CYP1A1 although changes were milder. 100 mg/kg b.w. treatment showed a 32% (*P* < 0.001; *n* = 5) increment after 7 days that became non-significant at the fourteenth day. The highest dose resulted in 74 and 46% increments after 7 and 14 days, respectively (*P* < 0.001; n = 5). (**d**) CYP1A1/2-ethoxyresorufin *O*-deethylase increased from 26% at the lowest dosage for 7 days to more than 100% after 14 days of treatment. The highest dose more than doubled at both time windows. CYP2B1/2-pentoxyresorufin *O*-dealkylase rose from 26 to 124% (*P* < 0.01; *n* = 6), depending on the dose when vitamin E was administered for 7 days, whereas changes became less marked (about 85% and 50%) after 14 days(*P* < 0.0; *n* = 6). (**e**) EPR in prostate tissue biopsies from vitamin E-injected rats (both doses and time frames) reported significantly (*P* < 0.05; *n* = 5) higher levels of radical species compared to controls. (**f**) Data from TBARS assay plot indicated a general trend toward oxidative stress in prostate tissue of vitamin E-treated animals, even if only the 14-day treatment (both doses) reached statistical significance (*P* < 0.05; *n* = 6). (**g**) Carbonylated proteins showed a sharp increment when vitamin E was administered for 7 days (*P* < 0.01; *n* = 9), whereas, extending the treatment to 14 days led to an almost full recovery. Bars represent the mean (±SD). All data were analyzed by the ANOVA test corrected for multiple comparisons (Sidak post-hoc). *P < 0.05; **P < 0.01; ***P < 0.001 (Control *vs* groups of each treatment).

To extend our analyses, we used a rat model to investigate whether vitamin E induces CYP expression and ROS generation *in vivo*. We observed that vitamin E treatment leads to increase in CYP1A1 gene expression in the prostate at all doses and timeframes considered, whereas CYP1A2 (activating dioxins, aromatic/heterocyclic amines, aflatoxins) and CYP1B1 (activating PAH, steroids) show a non-linear pathway of gene expression (Fig. 2a–c), the highest dose is associated to the more marked changes, whereas the low dosage shows a null effect after fourteen days (Fig. 2b,c) as well as a down-regulation in CYP1A2 at day seven (Fig. 2b). The corresponding enzyme activities here considered result boosted across all groups (Fig. 2d). These apparent inconsistencies could be explained considering the complexity of the CYP450 modulation phenomenon that can occur following different pathways involving numerous nuclear receptors as well as different dose-depending or time-depending mechanisms that can lead to a post-translational process increasing protein stability as well as transcriptional induction^26,27^. Moreover, a dose-response/time-response hormesis could be also considered as a putative explanation for our results^28^.

We hypothesize that corresponding high levels of CYPs predispose a subject to prostate cancer risk from the ubiquitous widely bioactivated pro-carcinogens^29,30^.

Furthermore, using the EPR spin-trapping technique, we observed that vitamin E in rat prostate generates high ROS levels irrespective of doses or timing (Fig. 2e) along with typical markers of oxidative stress injuries such as lipid peroxidation (Fig. 2f) and protein carbonylation (Fig. 2g). Even if not all vitamin E-exposed groups tested positive, it should be considered that malondialdehyde (MDA) as a marker of lipid peroxidation and the protein carbonyls here reported, were carried out in the microsomal and cytosolic fractions, respectively, and it is plausible to assume that different subcellular fractions could differently react to ROS injuries. Furthermore, the inducible antioxidant enzymatic machinery might have had a role in counteracting the oxidative stress in specific cellular compartments. However, the reactive oxygen species yield determined by EPR in biopsy gives a complete and accurate picture of ROS content in the cellular and extracellular matrix (Fig. 2e). As a whole these results support the hypothesis of ROS over generation by CYP induction in prostate tissue that can result in cell damage and inflammation^29^. Although our result on oxidative stress markers did not show a linear trend, in their entirety, they are consistent with those emerged from the trial by Pearson and colleagues in which an increment of plasma oxidation activity levels was observed in patients receiving vitamin E^31^.

**Figure 3:**
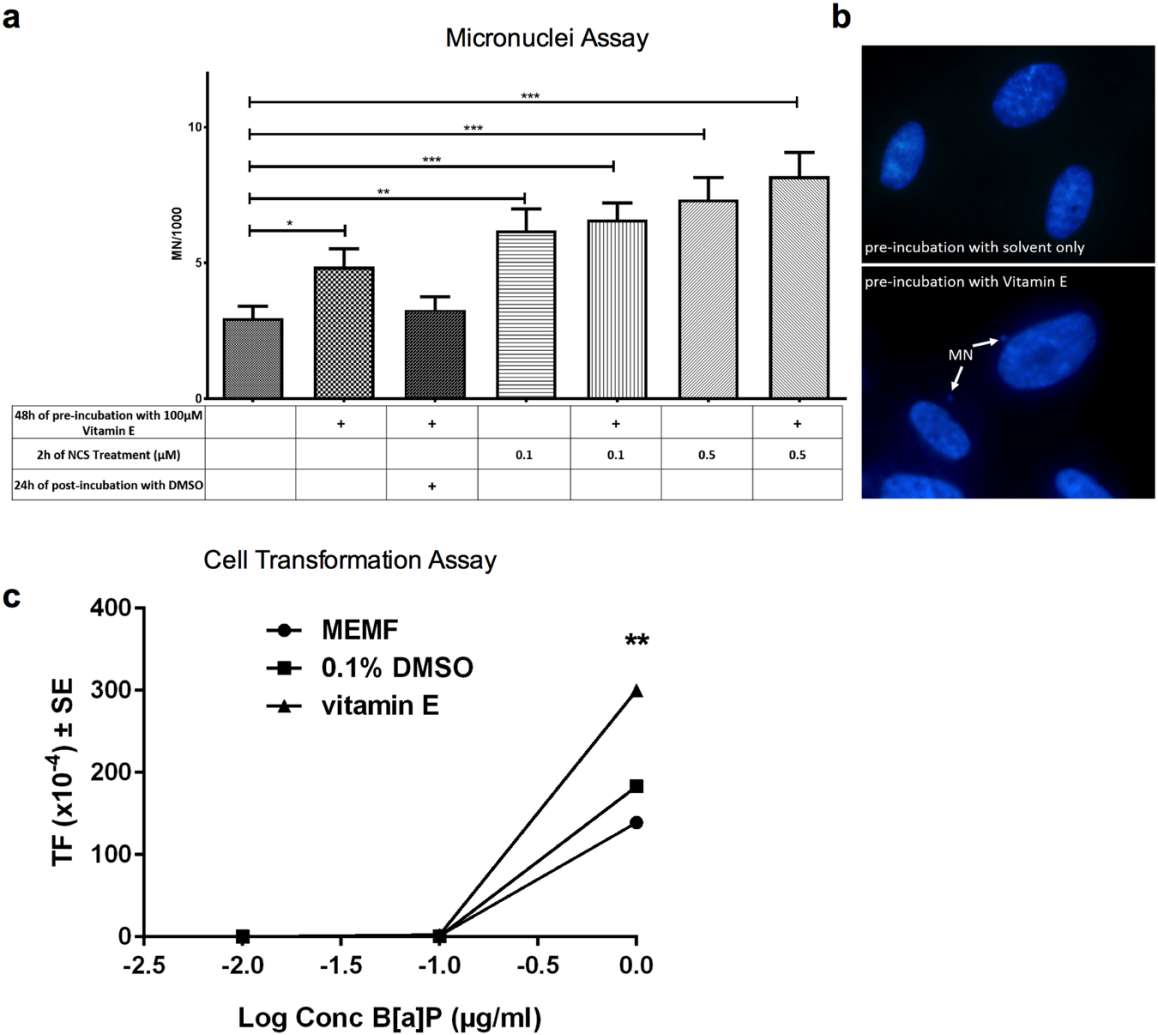
Vitamin E promotes DNA damage and cell transformation. (**a**) Human fibroblasts (IMR90) were treated with vitamin E (100 µM) and/or neocarzinostatin (NCS, used as positive control), 0.1 µM or 0.5 µM. DMSO was applied in control samples since it was used to dilute vitamin E. After 48 h of pre-incubation with vitamin E the cells were treated with NCS for 2 h and finally with MEM for 24 h (see Table below the histogram). The micronuclei were scored counting at least 1,000 nuclei per sample. A significance difference was observed between control and vitamin E-treated samples (*P* < 0.05) and between control and NCS-treated samples (*P* < 0.01 and *P* < 0.001). Bars represent the mean (±SE). All data were analyzed by the unpaired t-test *P < 0.05; **P < 0.01; ***P < 0.001. (**b**) An example of DAPI-stained nuclei of cells pre-incubated with DMSO (control, upper) and DMSO plus vitamin E (below). Arrows indicate micronuclei. (**c**) BALB/c3T3 cells were seeded for cellular transformation assay (CTA), maintained in culture for 24 h and exposed to minimum essential medium (MEM) supplemented with 10% fetal bovine serum (MEMF), 0.1% DMSO or 100 µM vitamin E for 24 h. Then cells were treated with B[a]P (final concentrations 0.01 - 0.1 - 1 µg/ml) for 48 h. Four weeks after seeding, cells were fixed, stained and scored for transformed foci. The type III foci number was determined in each plate, according to the established scoring criteria. Statistical analysis of foci distribution was performed by the Mann–Whitney unpaired t-test. The transformation frequency (TF) was calculated on the basis of the foci number divided by the clonal efficiency (ACE) observed in the cytotoxicity assay performed concurrently. ACE represented the number of cells surviving chemical treatment. Cells treated with vitamin E and then with B[a]P at concentrations leading to cell transformation showed a significant increase in TF (*P* < 0.01, Exact Poisson test). (Control *vs* groups of each treatment).

Collectively, our data strengthen the hypothesis that vitamin E could act as a co-carcinogenic agent by means of both its increased metabolic activating potential towards pro-mutagens/pro-carcinogens and its pro-oxidant activity and thus might drive tumorigenesis through DNA insults and epigenetic alterations. To test our hypothesis, we examined whether vitamin E induces DNA damage to histone variant H2AX and 53BP1 as an indirect measure of double strand DNA breaks (DSBs)^32^. Although ROS pronominally lead to a single strand DNA breaks, DSBs can result following replication past oxidative stress-induced lesions^33^. We observed the appearance of a small proportion of human fibroblasts displaying more than 20 foci per nucleus in vitamin E-treated cultures (Supplementary Fig. S1). Although differences did not reach statistical significance, they suggest that vitamin E may impair genomic stability. Thus we measured micronuclei (MN) abundance after vitamin E pre-incubation, finding that vitamin E worsens the genomic stability of cells by significantly increasing the number of MN and also slightly increasing the number of MN in neocarzinostatin (NCS)-treated positive control cultures (Fig. 3a,b). It is reasonable to suppose that the CYP induced during the pre-incubation of fibroblasts with vitamin E are responsible for the subsequent ROS generation leading to DNA damage occurring without vitamin E being present in the cultures. Indeed, cell incubation with DMSO, which has radical scavenging properties^34^, tends to restore the control level of MN, suggesting the possible oxidative nature of the damage (Fig. 3a).

Lastly, to test the co-carcinogenic properties of vitamin E directly, we used the BALB/c 3T3 *in vitro* cell transformation assay (CTA), which models key stages of *in vivo* carcinogenesis and has been proposed as an alternative to the rodent cancer bioassay^35^. Cells were pre-incubated with vitamin E, then treated with the ubiquitous environmental pollutant carcinogenic benzo[a]pyrene (B[a]P) selected as a PAH prototype. Vitamin E significantly enhanced the cell transformation frequency (TF) of B[a]P-transformed cells, whereas it did not induce cell transformation per se or prevent B[a]P-induced transformation (Fig. 3b).

These results are consistent with the observed inducing effect of vitamin E toward the CYP1A1 isoform, the main enzyme responsible for (B[a]P) activation. It can be argued that by boosting phase-I CYP bioactivating enzymes during cell growth, vitamin E may then trigger the conversion of (B[a]P) to ultimate more carcinogenic reactive intermediates such as diol-epoxides^12^.

In summary, this work demonstrates that vitamin E has a co-carcinogenic effect by boosting the enzymes that bioactivate the pro-carcinogens usually found up regulated in prostate tumours and advocated as promising targets for cancer treatment^12,13^. Our results are in line with those previously reported by McCormick and colleagues who found an increase in prostate cancer incidence in rats fed 4000 mg/kg vitamin E in the diet^36^. However, it is mandatory to point out that cell lines were exposed to approximately 2.5 fold higher vitamin E concentration compared to the amount (16 µg/mL) reached in the study by McCormick *et al*., as well as 100 and 200 mg/kg b.w. i.p. (16,5–33 mg/rat weighing 165 g) would result in a vastly higher circulating vitamin E levels compared to the rat feeding study quoted above^36^ and to patients from SELECT (<9–~20 μg/mL)^25^. Furthermore, we demonstrated that vitamin E generates a persistent oxidative stress, which in turn leads to a flare-up in inflammation recently associated with the prostate cancer risk in a prospective study combining data from the Prostate Cancer Prevention Trial (PCPT) and the SELECT cohorts^37^. On the other hand, oxidative stress is widely involved in the initiation and development of many malignancies, including prostate cancer^38^.

## Conclusions

Consistent with these mechanisms, our study also demonstrated that vitamin E promotes DNA damage and cell transformation, thereby providing a biological rationale for the paradoxical effect of the increased prostate cancer incidence among healthy men supplemented with vitamin E in the SELECT study^7^, even if we have to bear in mind that results here discussed were achieved exposing cell lines and animals to higher vitamin E concentrations compared to those reported in the SELECT. Other factors contributing to prostate cancer development by vitamin E cannot be excluded. Our study adds new evidence that some antioxidants exert a dichotomous suppressive and promoting activity with respect to tumorigenesis^39^.

The present work should not be intended as a translational study. The models here discussed had the only purpose to test the hypothesis of a co-carcinogenesis mechanism of vitamin E through CYP supergene family induction. The dosages here used are not referred to human consumption of vitamin E neither to those described in the SELECT; such a limitations should be considered.

## Methods

### Chemicals

The form of vitamin E considered in the study was DL-all-rac α-tocopherol (PubChem CID: 2116) purchased from Sigma Aldrich Chemicals Co (USA). Neocarzinostatin (NCS) was also from Sigma Aldrich Chemicals.

### Cell culture and exposure

The prostate epithelial cell line RWPE-1 (normal prostatic epithelial cells derived from histological normal adult prostate) was obtained from Dr. Catia Giovannini, Center for Applied Biomedical Research, St. Orsola-Malpighi University Hospital, Bologna, Italy. RWPE-1 was used and cultured in a standard 37 °C humidified incubator with 5% carbon dioxide and 95% oxygen. RWPE-1 was cultured in defined keratinocyte serum-free medium (KSFM) supplemented with human recombinant epidermal growth factor (rEGF) and bovine pituitary extract (BPE) (Gibco, USA). Cells were plated at a density of 5 × 10^5^ cells/well in 6-well plates and cultured until they reached a 50–60% confluence and then treated with vitamin E ((+)-α-tocopherol, C_29_H_5_O_2_) solubilized in dimethyl sulfoxide (DMSO) 0.1%. Vitamin E concentrations used for cell treatments ranged from 25 μM (comparable to physiological plasma levels) to 150 μM^40,41^, DMSO was also added in untreated control cells.

### Cell viability

Cell viability was measured by Sulphorodamine B assay (SRB assay, Sigma, USA) in according to the manufacture’s sheet. The absorbance was read at 570 nm. Vitamin E did not show cytotoxic activity against the RWPE-1 cell line, even at high doses, after 72 hours (data not shown).

### RNA extraction from RWPE-1 cells and gene expression analysis

Total RNA from vitamin E-treated (100 μM; 48 h) RWPE-1 cells was extracted using Trizol reagent (Life Technologies, CA, USA) in accordance to the manufacturer’s instructions. DNase I treatment (DNA-free kit, Ambion, USA) was adopted to remove any genomic DNA contamination. Extracted RNA samples were reverse-transcripted using using RevertAid™ First Strand cDNA Synthesis Kits (Fisher Scientific, KS, USA)^42^. CYPs, COX-2 and β-actin mRNA levels were analyzed by real-time PCR using SYBR- Select Master Mix (Life Technologies, CA, USA) and StepOne PlusTM system (Applied Biosystems, CA, USA). The melting curve data were collected to check PCR specificity. Each cDNA sample was analyzed in triplicate. Target mRNA levels were normalized against β-actin mRNA Relative expressions were calculated using the formula 2^−2ΔCt^ values (ΔCt = Ctgene − Cthk). Primers were purchased from IDT technologies (USA). All samples containing 200 ng of cDNA were run in triplicate. The thermal cycler was programmed as follows: 30 s at 95 °C and 40 cycles of 5 s at 95 °C and 20 s at 60 °C for amplification.

Primer sequences:

**β-actin**: F5′-GGCGGCACCACCATGTACCCT-3′; R5′-AGGGGCCGGACTCGTCATACT-3′;

**CYP1A1**: F5′-CAAGAGGAGCTAGACACAGT-3′; R5′-AGCCTTTCAAACTTGTGTCT-3′;

**CYP1A2**: F5′-GGACAGCACTTCCCTGAGAG-3′; R5′GAGGCAGTCTCCACGAACTC-3′;

**CYP3A5**: F5′-GCTCGCAGCCCAGTCAATA-3′; R5′-AGGTGGTGCCTTATTGGGC-3′;

**CYP1B1**: F5′-TTCGGCCACTACTCGGAGC-3′; R5′-AAGAAGTTGCGCATCATGCT-3′;

**CYP2B6**: F5′-TCCAGTCTCAGCTCCCAAGT-3′; R5′-CTGGCCAACATGTCCCTACT-3′;

**CYP2C9**: F5′-CCACATGCCCTACACAGATG-3′; R5′-TGCCCTTGGGAATGAGATAG-3′;

**CYP4F2**: F5′-GACACCAGGGCATGGTCAACC-3′; R5′-CGGCAATATATTCACTGGGTTTC-3′;

**COX-2:** F5′-CCTGTGCCTGATGATTGC-3′; R5′-CTGATGCGTGAAGTGCTG-3′.

### Electronic paramagnetic resonance (EPR) measurements

This method was previously employed as an accurate measure of total radical species in different tissues^43^. The biopsies are dissolved in a 1 mM solution of the hydroxylamine “spin trap” (bis(1-hydroxy-2,2,6,6-tetramethyl-4-piperidinyl) decandioate dihydrochloride CAS no. 314726-62-0) and incubated at 37 °C for 5 minutes. Samples from the solution are loaded in a capillary glass tube and inserted in the cavity of a Bruker ESP 300 EPR spectrometer (Bruker Biospin S.r.l., Rheinstetten, Germany). The nitoxide spectra were recorded at the following settings: modulation amplitude = 1.0 G; conversion time = 163.84 ms; time constant = 163.84 ms; modulation frequency 100 kHz; microwave power = 6.4 mW. All specifications about instrument calibration and data elaboration were previously reported^38^. Tests on RWPE-1 cells were carried out as the procedures described above: cells were seeded in a 6-well plate at a density of 7 × 10^5^ cells per well and collected in serum-free medium for 48 h with vitamin E or PBS. The medium was discarded and cells detached with a scraper. Cells were centrifuged at 300 × g for 5 min and suspended in 500 μL of PBS 1X + 500 μL of hydroxylamine “spin trap” (bis(1-hydroxy-2,2,6,6-tetramethyl-4-piperidinyl) decandioate dihydrochloride (1 mM final concentration).

### PGE2 measurement through ELISA tests

PGE2 in RWPE-1 supernatants was determined using the PGE2 ELISA assay (Cayman, Ann Arbour, USA). Briefly, cells were seeded in a 6-well plate at a density of 3 × 10^5^ cells per well in serum-free medium for 48 h in the presence or absence of vitamin E. The harvested media were centrifuged at 500 × g for 5 min (4 °C) to remove floating cells. Supernatants were finally assayed following the supplier’s instructions and the absorbance was measured by using a microplate reader (Bio-Rad, USA) at 570 nm.

### Animal treatment

The study was approved by the University of Bologna Committee on the Ethics of Animal Experiments and by the Italian Ministry of Health (Prot n. 43 IX-9). All experiments were performed in accordance with relevant guidelines and regulations. 8 week-old male Sprague-Dawley rats weighing 150–180 g were purchased from ENVIGO RMS S.r.l. (San Pietro al Natisone, Udine, Italy). They were housed under a 12h-light/12h-dark cycle, 22 °C, 60% humidity, and fed ad libitum with a standard rodent chow with a total amount of vitamin E around 123 mg/kg (Mucedola Srl). After an adequate period of acclimatization animals were randomly divided into 6 experimental units of 6 animals each. Vitamin E was dissolved in corn oil for fat-soluble compounds by Sigma-Aldrich Merck group and administered intraperitoneally (i.p.) at 100 or 200 mg/kg b.w. for 7 or 14 consecutive days. Controls received an equal volume of vehicle. The present work was not conceived as a translational study; therefore it does not intend to simulate a putative human intake. Dosages were based on literature review^36,44,45^.

### Tissue collection and subcellular fractions

Animals were injected with Zoletil 100 (100 mg/kg b.w.) and then scarified according to the Ministerial procedures. The prostate was resected frozen in liquid nitrogen. Microsomes and cytosolic fraction were prepared as previously reported^20^.

### mRNA extraction from prostate tissue and gene expression analysis

Total RNA was extracted from tissue by the use of the RNeasy Mini Kit (Qiagen, Valencia, CA, USA) following the manufacturer’s sheet and then quantified using NanoDrop (Celbio). Purity and integrity levels were checked monitoring the absorbance ratios A_260_/A_280_ nm and A_260_/_230_ and assessing the sharpness of 18S and 28S ribosomal RNA bands on 1% agarose gel stained with GelRed™ (Biotium, Hayward, CA). Genomic DNA elimination and reverse transcription of total RNA were performed using the QuantiTech Reverse Transcription Kit (Qiagen). Full details on procedure were previously reported^46^. β-actin was utilized as endogenous control to normalize for RNA loading or differences in reverse transcription efficiency.

Primer sequences:

**CYP1A1**: F5′-CTTGCAAAGCCCATGTTCCT-3′; R5′-TGGTGTAGCGGTTCATGACT-3′

**CYP1A2**: F5′-TGGTGGAATCGGTGGCTAAT-3′; R5′-TAAACCTCTTGAGGGCTGGG-3′

**CYP1B1:** F5′- GGTGGCTAATGTCATGAGCG-3′; R5′-AAGTTGCTGAAGTTGCGGTT-3′.

Data about gene expression discussed above were obtained through different RNA extraction methods. This represents a potential limitation of the study.

### CYP1A1/2 and CYP2B1/2 enzyme activities

All enzymatic assays and protein concentrations were performed as previously described^47^.

### Lipid peroxidation and protein carbonylation

Protein carbonyl groups: the assay was performed in accordance with the method described by Levine and colleagues^48^ with slight changes. The carbonyl groups were determined following spectrophotometrically their covalent reaction with 2,4-dinitrophenylhydrazine (DNPH), that leads to the formation of a yellow 2,4-dinitrophenyl (DNP) hydrazone by-product. The cytosolic proteins were precipitated with cold trichloroacetic acid (TCA) 20% and then collected by 10 min centrifugation (4,000 g). A solution of 10 mM DNPH in 2 N HCl was added to the protein. Blanks were added with only 2 N HCl. Samples were incubated for 1 hour at room temperature vortexing every 10 min and then precipitated with TCA 20% and centrifuged. Pellets were washed three times with a solution of ethanol and ethyl acetate (1:1 v/v) and suspended in PBS buffer (pH 2,3). All samples were centrifuged (4,000 rpm) and the absorbance of the supernatant was read at 390 nm (ε = 22 mM^−1^).

Malonyldialdehyde (MDA) levels: MDA levels in the microsomal fraction were determined according to the method of Esterbauer and Zollner^49^. Samples were diluted in a solution with 1 mL acetic acid 20%, 1.5 mL of thiobarbituric acid (TBA) 0.8%, 0.2 mL of sodium dodecyl sulfate (SDS) 8%. The mixture was heated in a boiling water bath at 95 °C for 1 h. A calibration curve was carried out using 1,1,3,3-tetramethoxypropane dissolved in water and properly diluted. Absorbance was read at 535 nm. Results were expressed as moles of MDA h^−1^ mg protein^−1^.

### Micronuclei assay

Human fibroblasts (IMR90) were maintained in minimum essential medium (MEM) with Earle’s Salts and L-glutamine containing 10% fetal bovine serum (FBS), MEM vitamins and amino acids, and penicillin-streptomycin (all from Sigma Aldrich, St. Louis, MO, USA). Cells were seeded onto glass coverslips at a density of 10,000 cells for cm^2^ and treated with vitamin E (100 μM) and NCS 0.1 µM and 0.5 µM (from Sigma Aldrich, St. Louis, MO, USA). Since vitamin E was diluted in DMSO, same amounts of DMSO were applied in control samples. After pre-incubation with vitamin E for 48 h, the cells were rinsed twice and treated with NCS (0.1 μM and 0.5 μM) for 2 h, after two additional washes the cells were finally incubated for 24 h in MEM with or without DMSO. Cells were fixed with 4% paraformaldehyde, permeabilized in 0.2% Triton X100 in PBS, stained with DAPI (1 μL of DAPI in 5 mL of 0.1% PBS-BSA) and mounted with Vectashield Mounting Medium (Vector Laboratories, Burlingame, CA, USA) before analysis with an Olympus IX50 fluorescence microscope equipped with a Canon camera.

Micronuclei, defined as DAPI-positive bodies that were morphologically identical to but smaller than the nucleus, were scored counting at least 1,000 nuclei per slide.

### Immunofluorescence determination of 53BP1 and γH2AX

On IMR90 human fibroblasts cultured as previously described in the micronucleus assay section and unsynchronized, DNA damage foci were scored following immunofluorescent staining performed as previously described^33,50^. Nuclei were scored as containing: γH2AX and 53BP1 foci (F): F ≤ 5; 5 < F ≤ 20; F > 20.

### Cell transformation assay

The original stock of BALB/c 3T3 cells, clone A31-1-1, was obtained from the Health Science Research Resource Bank (Osaka, Japan). Cells were grown in minimum essential medium (MEM) supplemented with 10% fetal bovine serum (FBS, Gibco BRL) and maintained in a humidified incubator with an atmosphere of 5% CO_2_ in air at 37 °C. For the cell transformation assay (CTA), sub-confluent cells were seeded at a density of 3 × 10^4^ cells/2 ml/60-mm plate, 10 replicates for each treatment. At 24 h after seeding, cells were treated with vitamin E. The stock solution of vitamin E (1 M in DMSO) was diluted 1:10 in DMSO and then in MEM, to obtain a 300 µM working solution. The final concentration of vitamin E (100 µM) was obtained by 1:3 dilution of the 300 µM solution directly into cell culture plates. Untreated cells and DMSO-treated cells received 1 ml of MEM or 0.1% DMSO solution, respectively. At 48 h after seeding, the treatment solutions were removed and replaced with medium containing benzo[a] pyrene (B[a]P, CAS number 50-32-8, purity 96%, Ultra Scientific Italia). The working solutions of B[a]P were prepared by 1:1,000 dilution of the DMSO stock solutions immediately before use. The final concentrations ranged from 1 µg/ml to 0.01 µg/ml. Positive controls were represented by cells treated with 4 µg/ml 3-methylcholanthrene (MCA, CAS number 56-49-5, purity 99%, Ultra Scientific Italia). The working solution was prepared by diluting MCA in DMSO at 4 mg/ml and then 1:1,000 in M10F. DMSO (CAS number 67-68-5), which was used as the solvent vehicle for all the chemicals, was administered to cell cultures at a final concentration of 0.1%. At 96 h after seeding, the treatment solutions were removed and replaced with MEM. Cells were maintained in culture for 4 weeks with twice weekly medium changes, then fixed with methanol, stained with 10% aqueous Giemsa and scored for foci formation. The foci hallmarks for the inclusion were detailed reported previously^35^.

### Statistical analysis

Data sets on RWPE-1 cells were analyzed using the two-tailed t-test. Data from *in vivo* experiments were analyzed using the ANOVA test with Sidak correction for multiple comparisons. Data from micronuclei assay and immunofluorescence determination of 53BP1 and γH2AX were analyzed using the unpaired t-test. Data from transformation assay were analyzed by the one tailed Mann–Whitney unpaired t-test. TF statistical significance was assessed by comparing the Poisson rates^51^. Unless otherwise indicated, data from each group were compared with relative control. *P < 0,05; **P < 0,01; ***P < 0,001.

## Supplementary information

Supplementary Figure S1

## References

[1] Cuzick J (2017). Preventive therapy for cancer. Lancet Oncol..

[2] Chikara S (2018). Oxidative stress and dietary phytochemicals: Role in cancer chemoprevention and treatment. Cancer Lett..

[3] Hampton T (2005). Clinical trials point to complexities of chemoprevention for cancer. JAMA..

[4] Lippman SM (2009). Effect of selenium and vitamin E on risk of prostate cancer and other cancers: the Selenium and Vitamin E Cancer Prevention Trial (SELECT). JAMA..

[5] Stratton J, Godwin M (2011). The effect of supplemental vitamins and minerals on the development of prostate cancer: a systematic review and meta-analysis. Fam Pract..

[6] Bosland MC (2016). Is There a Future for Chemoprevention of Prostate Cancer?. Cancer Prev Res (Phila)..

[7] Klein EA (2011). Vitamin E and the risk of prostate cancer: the Selenium and Vitamin E Cancer Prevention Trial (SELECT). JAMA.

[8] Ballon-Landa E, Parsons JK (2018). Nutrition, physical activity, and lifestyle factors in prostate cancer prevention. Curr Opin Urol..

[9] Njoroje RN (2017). Organoids model distinct Vitamin E effects at different stages of prostate cancer evolution. Sci Rep..

[10] Chang I (2018). Cytochrome P450 1B1 inhibition suppresses tumorigenicity of prostate cancer via caspase-1 activation. Oncotarget..

[11] Zhu W (2019). Associations of CYP1 polymorphisms with risk of prostate cancer: an updated meta-analysis. Biosci Rep..

[12] Mitsui Y (2016). Functional role and tobacco smoking effects on methylation of CYP1A1 gene in prostate cancer. Oncotarget..

[13] D’Uva G, Baci D, Albini A, Noonan DM (2018). Cancer chemoprevention revisited: Cytochrome P450 family 1B1 as a target in the tumor and the microenvironment. Cancer Treat Rev..

[14] Cheng J (2014). Vascular characterization of mice with endothelial expression of cytochrome P450 4F2. FASEB J..

[15] Vanella L (2013). Effects of ellagic acid on angiogenic factors in prostate cancer cells. Cancers (Basel)..

[16] Paolini M, Biagi GL, Cantelli-Forti G, Bauer C (1994). Further mechanisms of non-genotoxic carcinogenesis. Trends Pharmacol Sci..

[17] Stiborová M (2018). Exposure to endocrine disruptors 17alpha-ethinylestradiol and estradiol influences cytochrome P450 1A1-mediated genotoxicity of benzo[a]pyrene and expression of this enzyme in rats. Toxicology..

[18] Paolini M (1996). Paramagnetic resonance in detecting carcinogenic risk from cytochrome P450 overexpression. J Investig Med..

[19] Veith A, Moorthy B (2017). Role of cytochrome p450s in the generation and metabolism of reactive oxygen species. Curr Opin Toxicol..

[20] Vivarelli F (2016). Disruption of redox homeostasis and carcinogen metabolizing enzymes changes by administration of vitamin E to rats. Life Sci..

[21] Kochel TJ, Reader JC, Ma X, Kundu N, Fulton AM (2017). Multiple drug resistance-associated protein (MRP4) exports prostaglandin E2 (PGE2) and contributes to metastasis in basal/triple negative breast cancer. Oncotarget..

[22] Tong D (2017). Metformin inhibits castration-induced EMT in prostate cancer by repressing COX2/PGE2/STAT3 axis. Cancer Lett..

[23] Brizzolara A (2017). The ErbB family and androgen receptor signaling are targets of Celecoxib in prostate cancer. Cancer Lett..

[24] Turanli, B. *et al*. Drug Repositioning for Effective Prostate Cancer Treatment. *Front Physiol*. **500**, eCollection 2018 (2018). 10.3389/fphys.2018.00500 PMC5962745 29867548

[25] Albanes D (2014). Plasma tocopherols and risk of prostate cancer in the Selenium and Vitamin E Cancer Prevention Trial (SELECT). Cancer Prev Res (Phila)..

[26] Fernandez-Abascal J, Ripullone M, Valeri A, Leone C, Valoti M (2018). β-Naphtoflavone and Ethanol Induce Cytochrome P450 and Protect towards MPP^+^ Toxicity in Human Neuroblastoma SH-SY5Y Cells. Int J Mol Sci..

[27] Hu Y, Ingelman-Sundberg M, Lindros KO (1995). Induction mechanisms of cytochrome P450 2E1 in liver: interplay between ethanol treatment and starvation. Biochem Pharmacol..

[28] Calabrese EJ, Mattson MP (2017). How does hormesis impact biology, toxicology, and medicine?. NPJ Aging Mech Dis..

[29] He X, Feng S (2015). Role of Metabolic Enzymes P450 (CYP) on Activating Procarcinogen and their Polymorphisms on the Risk of Cancers. Curr Drug Metab..

[30] Sapone A (2012). On enzyme-based anticancer molecular dietary manipulations. J Biomed Biotechnol..

[31] Pearson P, Lewis SA, Britton J, Young IS, Fogarty A (2006). The pro-oxidant activity of high-dose vitamin E supplements *in vivo*. BioDrugs..

[32] Croco E (2017). DNA Damage Detection by 53BP1: Relationship to Species Longevity. J Gerontol A Biol Sci Med Sci..

[33] Woodbine L, Brunton H, Goodarzi AA, Shibata A, Jeggo PA (2011). Endogenously induced DNA double strand breaks arise in heterochromatic DNA regions and require ataxia telangiectasia mutated and Artemis for their repair. Nucleic Acids Res..

[34] Lodovici M (2001). Antioxidant and radical scavenging properties *in vitro* of polyphenolic extracts from red wine. Eur J Nutr..

[35] Mascolo MG (2010). BALB/c 3T3 cell transformation assay for the prediction of carcinogenic potential of chemicals and environmental mixtures. Toxicol. In Vitro..

[36] McCormick DL (2010). Null activity of selenium and vitamin e as cancer chemopreventive agents in the rat prostate. Cancer Prev Res (Phila)..

[37] Platz EA (2017). A Prospective Study of Chronic Inflammation in Benign Prostate Tissue and Risk of Prostate Cancer: Linked PCPT and SELECT Cohorts. Cancer Epidemiol Biomarkers Prev..

[38] Prasad S, Gupta SC, Tyagi AK (2017). Reactive oxygen species (ROS) and cancer: Role of antioxidative nutraceuticals. Cancer Lett..

[39] Propac P (2017). Targeting Free Radicals in Oxidative Stress-Related Human Diseases. Trends Pharmacol Sci..

[40] Campbell SE (2006). Comparative effects of RRR-alpha- and RRR-gamma-tocopherol on proliferation and apoptosis in human colon cancer cell lines. BMC Cancer..

[41] Huang H (2014). Potent Inhibitory Effect of δ-Tocopherol on Prostate Cancer Cells Cultured *in Vitro* and Grown As Xenograft Tumors *in Vivo*. J. Agric. Food Chem..

[42] Pavan B (2018). Geraniol Pharmacokinetics, Bioavailability and Its Multiple Effects on the Liver Antioxidant and Xenobiotic-Metabolizing Enzymes. Front. Pharmacol..

[43] Fabbri R (2015). Effects of N-acetylcysteine on human ovarian tissue preservation undergoing cryopreservation procedure. Histol Histopathol..

[44] Mustacich DJ, Leonard SW, Devereaux MW, Sokol RJ, Traber MG (2006). Alpha-tocopherol regulation of hepatic cytochrome P450s and ABC transporters in rats. Free Radic Biol Med..

[45] Traber MG, Labut EM, Leonard SW, Lebold KM (2011). α-Tocopherol injections in rats up-regulate hepatic ABC transporters, but not cytochrome P450 enzymes. Free Radic Biol Med..

[46] Vornoli A, Pozzo L, Della Croce CM, Gervasi PG, Longo V (2014). Drug metabolism enzymes in a steatotic model of rat treated with a high fat diet and a low dose of streptozotocin. Food Chem Toxicol..

[47] Bonamassa B (2016). Harmful effects behind the daily supplementation of a fixed vegetarian blend in the rat model. Food Chem Toxicol..

[48] Levine RL (1990). Determination of carbonyl content in oxidatively modified proteins. Methods Enzymol..

[49] Esterbauer H, Zollner H (1989). Methods for determination of aldehydic lipid peroxidation products. Free Radic Biol Med..

[50] Canistro (2017). E-cigarettes induce toxicological effects that can raise the cancer risk. Sci Rep..

[51] Vaccari M (2015). Identification of pathway-based toxicity in the BALB/c 3T3 cell model. Toxicol In Vitro..

