# Supplementary material for: Co-carcinogenic effects of vitamin E in prostate
Source: Sci Rep. 2019 Aug 12;9:11636. doi: 10.1038/s41598-019-48213-1 (PMC6690912; doi:10.1038/s41598-019-48213-1)
Supplement: Supplementary file 1 — Supplementary Figure S1 [file 41598_2019_48213_MOESM1_ESM.docx]

**Supplementary information**

Co-carcinogenic effects of vitamin E in prostate

Fabio Vivarelli^1*^, Donatella Canistro^1*^, Silvia Cirillo^1^, Alessio Papi^2^, Enzo Spisni^2^, Andrea Vornoli^3^, Clara M. Della Croce^4^, Vincenzo Longo^4^, Paola Franchi^5^, Sandra Filippi^6^, Marco Lucarini^5^, Cristina Zanzi^7^, Francesca Rotondo^8^, Antonello Lorenzini^9^, Silvia Marchionni^9^ & Moreno Paolini^1^

***These authors contributed equally to this work**

Fabio Vivarelli & Donatella Canistro

**Corresponding author**

Correspondence to: Donatella Canistro (donatella.canistro@unibo.it)

**Affiliations**

^1^Department of Pharmacy and Biotechnology, University of Bologna, Bologna, Italy.

^2^Department of Biological, Geological and Environmental Sciences, University of Bologna, Bologna, Italy.

^3^Cesare Maltoni Cancer Research Center (CMCRC), Ramazzini Institute (RI), Bentivoglio, Bologna, Italy.

^4^Department of Chemistry "G. Ciamician", University of Bologna, Bologna, Italy.

^5^Department of Agricultural Biology and Biotechnology, CNR, Pisa, Italy

^6^Interdepartmental Laboratory of Functional and Cellular Pharmacology of Reproduction, Department of Neuroscience, Drug Research and Child Care, University of Florence, Florence, Italy.

^7^Center for Environmental Toxicology, Environmental Protection and Health Prevention Agency Emilia-Romagna Region (ER-EPA), Bologna, Italy.

^8^Center for Environmental Toxicology, Agency for Prevention, Environment and Energy, Emilia-Romagna, Bologna, Italy.

^9^Department of Biomedical and Neuromotor Sciences, University of Bologna, Bologna, Italy.


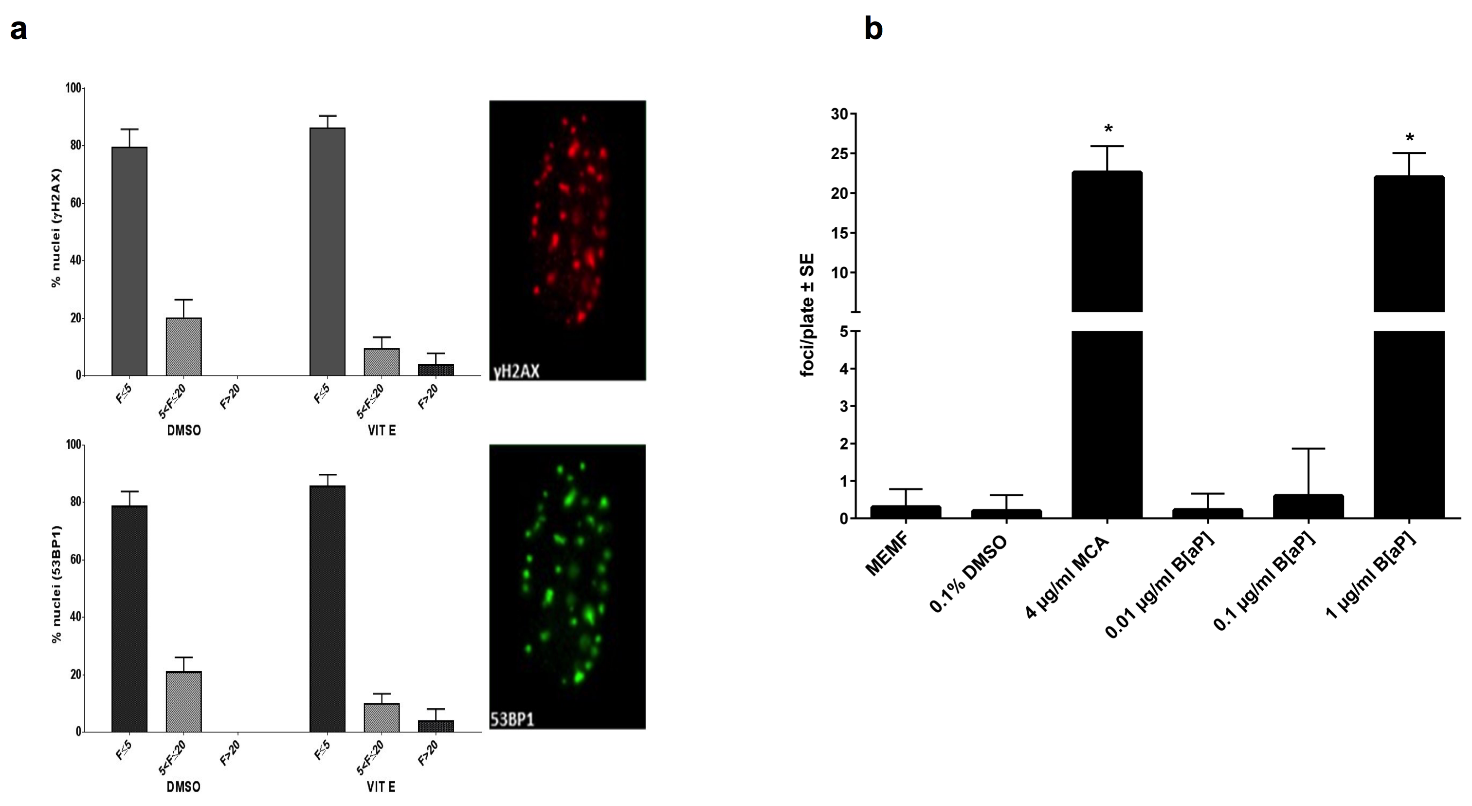


**Supplementary figure S1 Legend**

**a**, Left: percentage of DNA damage foci of H2AX (top histogram) and 53BP1 (bottom histogram). The number of foci (F) was scored as F ≤ 5; 5 < F ≤ 20; F > 20. All data were analyzed by the unpaired t-test. Right: nuclei of cells pre-incubated with vitamin E (VIT E) and immunostained for γH2AX (top figure) and 53BP1 (bottom figure). These nuclei are representative of cells with >20 foci. Bars represent the mean (± SE). All data were analyzed by the unpaired t-test. **P*<0.05; ***P*<0.01; *** *P*<0.001**. b**, Cells were seeded at a density of 3 x 10^4^ cells/2 ml/60-mm plate, 10 replicates for each treatment and incubated at 37 °C in 5% CO_2_ humidified air. At 48 h after seeding, cells were treated with B[a]P. At 96 h after seeding, the treatment solutions were removed and replaced with MEMF. Cells were maintained in culture for 4 weeks with twice weekly medium changes, then fixed with methanol, stained with 10% aqueous Giemsa and scored for foci formation. The positive control 3-MCA (4 µg/ml) induced a statistically significant increase in the number of transformed type III foci, which were almost absent in untreated and solvent (DMSO 0.1%)-treated cells. B[a]P treatment induced cell transformation in BALB/c 3T3 A31-1-1 cells, with a significant increase in foci number after 1 µg/ml exposure (**P*<0.01, Mann–Whitney unpaired t-test).
